# Prediction of Selective Serotonin Reuptake Inhibitor Response Using Diffusion-Weighted MRI

**DOI:** 10.3389/fpsyt.2013.00005

**Published:** 2013-03-06

**Authors:** Christine DeLorenzo, Lauren Delaparte, Binod Thapa-Chhetry, Jeffrey M. Miller, J. John Mann, Ramin V. Parsey

**Affiliations:** ^1^Department of Psychiatry and Behavioral Science, Stony Brook UniversityStony Brook, NY, USA; ^2^Department of Psychiatry, Columbia UniversityNew York, NY, USA; ^3^Division of Molecular Imaging and Neuropathology, New York State Psychiatric InstituteNew York, NY, USA; ^4^Department of Radiology, Columbia UniversityNew York, NY, USA

**Keywords:** diffusion-weighted MRI, major depressive disorder, tractography, amygdala, hippocampus, treatment prediction, selective serotonin reuptake inhibitors

## Abstract

Pre-treatment differences in serotonergic binding between those who remit to antidepressant treatment and those who do not have been found using Positron Emission Tomography (PET). To investigate these differences, an exploratory study was performed using a second imaging modality, diffusion-weighted MRI (DW-MRI). Eighteen antidepressant-free subjects with Major Depressive Disorder received a 25-direction DW-MRI scan prior to 8 weeks of selective serotonin reuptake inhibitor treatment. Probabilistic tractography was performed between the midbrain/raphe and two target regions implicated in depression pathophysiology (amygdala and hippocampus). Average fractional anisotropy (FA) within the derived tracts was compared between SSRI remitters and non-remitters, and correlation between pre-treatment FA values and SSRI treatment outcome was assessed. Results indicate that average FA in DW-MRI-derived tracts to the right amygdala was significantly lower in non-remitters (0.55 ± 0.04) than remitters (0.61 ± 0.04, *p* < 0.01). In addition, there was a significant correlation between average FA in tracts to the right amygdala and SSRI treatment response. These relationships were found at a trend level when using the left amygdala as a tractography target. No significant differences were observed when using the hippocampus as target. These regional differences, consistent with previous PET findings, suggest that the integrity and/or number of white matter fibers terminating in the right amygdala may be compromised in SSRI non-remitters. Further, this study points to the benefits of multimodal imaging and suggests that DW-MRI may provide a pre-treatment signature of SSRI depression remission at 8 weeks.

## Introduction

Treatment selection for Major Depressive Disorder (MDD) is a process of trial and error since there are currently no clinical predictors of treatment efficacy on an individual level (Taylor et al., 2005; Leuchter et al., 2010). To reduce the morbidity and mortality associated with MDD, there is a critical need to identify such biomarkers (Taylor et al., 2005; Leuchter et al., 2010). Due to the role of the serotonergic system in the pathophysiology of depression (Meltzer, 1990; Ressler and Nemeroff, 2000) and the fact that the most common first-line antidepressants, selective serotonin reuptake inhibitors (SSRIs), target the serotonin transporter (Leuchter et al., 2008), an index of serotonergic health may provide this prognostic indicator (Miller et al., 2008, 2012a; Tenke et al., 2010). As a promising example of this, lower pre-treatment serotonin transporter (5-HTT) binding in the amygdala, midbrain, and anterior cingulate, as assessed using positron emission tomography (PET), has been associated with non-remission of MDD after 1 year of open naturalistic treatment (Miller et al., 2008). Similarly, lower pre-treatment 5-HT_1A_ autoreceptor binding in the raphe nucleus, the region from which most serotonergic neurons originate (Cook et al., 2006; Liu et al., 2010), has been associated with non-remission of MDD after 8 weeks of SSRI treatment (Miller et al., 2012a).

To better understand whether these indices can predict antidepressant response, it may be helpful to examine the neurobiological basis of the serotonergic binding differences between those who remit to treatment (remitters) and those who do not (non-remitters). In particular, the reportedly lower pre-treatment 5-HT_1A_ and 5-HTT binding in non-remitters may be due to either diminished health and/or number of serotonergic fibers or reduced 5-HT_1A_ expression. These possibilities may be examined using Diffusion-Weighted MRI Imaging (DW-MRI), which measures the diffusion of water molecules to assess imaged tissue properties (Beaulieu, 2002). Using DW-MRI, estimates of the directionality of water diffusion, called fractional anisotropy (FA), can be calculated at each voxel. FA values range from zero (isotropic diffusion) to one (anisotropic diffusion; Ressler and Nemeroff, 2000) and have been used as an index of the health of the identified fiber (white matter) tracts, with higher FA potentially reflecting a parallel organization of axons and greater myelination (Beaulieu, 2002; Sexton et al., 2009). In addition, DW-MRI can be used to identify fiber trajectory and number between a seed and target region.

Several studies have used DW-MRI to examine abnormalities in white matter associated with mood disorders (Sexton et al., 2009). A 2009 review of DW-MRI studies reported that a significant reduction in FA was observed in the frontal lobes of depressed subjects in six of the seven studies that examined that region (Sexton et al., 2009). Three of those studies also found lower FA in the temporal lobes (Sexton et al., 2009). A further meta-analysis examination of voxel-wise FA studies found a consistent effect of decreased white matter FA in the superior longitudinal fasciculus that correlated with number, intensity, and treatment history of depressive episodes (Murphy and Frodl, 2011). Similarly, it has been shown that lower FA in the inferior frontal brain region correlates with greater severity of depression symptoms in late-life depression (Nobuhara et al., 2006).

Based on the above studies, it is clear that trends are beginning to emerge regarding DW-MRI-measured changes in depressed individuals. However, a clinically useful measure has yet to be derived from this modality. On that front, combining PET-based measures with DW-MRI identified fiber tracts can enhance the information provided by both modalities. Due to the complex nature of depression and, more specifically, antidepressant treatment response, it is helpful to use such a multimodal approach to probe observed neurobiological differences (Sexton et al., 2009). Therefore, in this study, the basis of previous serotonergic PET findings was examined by identifying the location and health of DW-MRI-derived tracts between the raphe/midbrain and two target regions: the amygdala and hippocampus.

The amygdala was chosen as a target since dysfunction in amygdala circuitry has been reported in depression and anxiety (LeDoux, 2003; Holmes, 2008; Lowry et al., 2008; Jasinska et al., 2012). Additionally, as stated above, pre-treatment 5-HTT amygdala binding differences were found in antidepressant remitters compared with non-remitters (Miller et al., 2012a). The hippocampus has also been implicated in depression and antidepressant action (Lopez et al., 1998; McEwen and Magarinos, 2001; Jabeen Haleem, 2011) although we have not found pre-treatment serotonergic binding differences (using PET) in this region.

The DW-MRI analysis was performed on a subset of MDD subjects whose pre-treatment serotonergic expression was examined using PET (Miller et al., 2012a). These subjects were therefore investigated, prior to SSRI treatment, with two modalities. Using this multimodal approach may afford a richer understanding of potential antidepressant treatment response biomarkers, which would provide clinicians much needed guidance in choosing effective treatment strategies.

## Materials and Methods

### Subject inclusion/exclusion criteria

#### Depressed subjects

This study was approved by the Institutional Review Board of the New York State Psychiatric Institute. All subjects were included after providing informed consent. Subjects with DSM-IV MDD were recruited through online and print advertisements, as well as through referrals from neighboring outpatient clinics as part of an SSRI prediction treatment study using PET. In that study, PET imaging was performed using two tracers, [^11^C]WAY-100635 (Mathis et al., 1994) and [^11^C]DASB (Houle et al., 2000), on the same day prior to SSRI treatment (Miller et al., 2012a,b). Of the 24 subjects with MDD reported in that study, 18 also received a 25-direction diffusion-weighted MR image prior to treatment (within 1 week of the PET scans). These 18 subjects are described in Table 1. As previously outlined (Miller et al., 2012a), study eligibility was assessed by psychiatric and medical history, chart review, physical examination, routine blood tests, pregnancy test, and urine toxicology. Inclusion criteria consisted of: (1) age 18–65; (2) ability to provide informed consent; (3) meeting the DSM-IV criteria for MDD and in a current major depressive episode; (4) 17-item Hamilton Depression Rating Scale (HAM-D) score greater than or equal to 15; (5) general health and absence of unstable medical conditions; and (6) for subjects currently taking antidepressants: lack of benefit after trial of adequate dose and duration. Exclusion criteria included: (1) Alcohol or substance use disorder within 6 months of scan; (2) other current or past major psychiatric disorders including bipolar disorder, schizophrenia, schizoaffective illness, or psychotic disorders (comorbid anxiety disorders allowed), anorexia nervosa or bulimia nervosa in the past year; (3) for subjects < 33 years old, a first-degree family history of schizophrenia (to exclude individuals possibly presenting with the prodrome of schizophrenia); (4) inability to discontinue psychotropic drugs that may interfere with 5-HTT or 5-HT_1A_ receptors for at least 3 weeks (6 weeks for fluoxetine) prior to scanning, or history of significant decompensation during medication washout; (5) pregnancy, current lactation, or plans to conceive during the course of study participation; (6) use of any anti-coagulant/anti-platelet treatment with the exception of aspirin within 10 days; (7) IV drug or ecstasy use within the past 5 years; (8) dementia; (9) prior history of head trauma; (10) lack of response to >2 trials of antidepressant monotherapy of adequate dose and duration; (11) active suicidal ideation warranting inpatient admission or requiring immediate treatment intervention; (12) metal implants; (13) current or past exposure to radiation; or (14) lifetime history of glaucoma.

**Table 1:** **Subject clinical and demographic information**.

	Non-remitters (*n* = 10)	Remitters (*n* = 8)	*p*-value
Age	31.2 ± 11.3	34.3 ± 14.6	0.61
Baseline Hamilton (24-item)	24.4 ± 4.4	25.5 ± 7.1	0.69
Final Hamilton (24-item)	16.3 ± 2.5	5.1 ± 3.1	0.00
Duration of treatment (days)	65.1 ± 13.7	65.8 ± 5.4	0.90
Beck depression inventory	26.9 ± 8.5	23.8 ± 11.6	0.52
Beck hopelessness inventory	9.6 ± 5.0	7.6 ± 9.9	0.63
Lifetime aggression	16.0 ± 4.0	13.3 ± 2.5	0.38
Age of first depressive episode	23.6 ± 10.8	17.6 ± 2.5	0.15
# Females (%)	7 (70%)	4 (50%)	0.63*
# Suicide attempters (%)	4 (40%)	1 (13%)	0.31*

*p-value calculated by Fisher’s exact test.

### Study protocol

To reduce any confounds associated with multiple drug targets, a study drug highly selective for the serotonin transporter was needed. Escitalopram was therefore chosen because it is the most selective SSRI available (Owens and Rosenbaum, 2002). Additionally, escitalopram has been shown to have equal or greater effectiveness than other available SSRIs as well as favorable tolerability (Kirino, 2012).

All subjects were antidepressant-free for at least 23 days prior to imaging. Following baseline imaging, treatment was initiated with escitalopram at a dose of 10 mg daily for the first 4 weeks. At 4 weeks, escitalopram non-responders (those with <50% reduction in 24-item HDRS) had escitalopram increased to 20 mg. At 6 weeks, escitalopram non-remitters who were still taking 10 mg had escitalopram increased to 20 mg. Remission, assessed at approximately 8 weeks, was defined as a final 24-item HDRS < 10 and ≥50% reduction in HDRS from baseline.

### Imaging techniques

#### Anatomical

Anatomical magnetic resonance images (MRIs) were acquired on a 3.0T Signa Advantage system (GE Healthcare, Waukesha, WI, USA), as previously described (Ogden et al., 2007).

Each subject’s MRI was processed using the freely available Freesurfer software^1^. This parcellation (using the Desikan Killiany atlas) provided anatomical delineation of multiple regions including the midbrain, left and right amygdala, and left and right hippocampus.

#### Diffusion imaging

Diffusion-weighted MRI scans were acquired using a single-shot echo planar imaging (EPI) sequence. Scan parameters were as follows: TR (repetition time): 14000 ms, TE (echo time): 83 ms, Flip Angle: 90°, slice thickness: 3 mm, FOV (field of view): 240 mm × 240 mm, voxel dimensions: 0.94 mm × 0.94 mm ×  3 mm, acquisition matrix: 132 × 128, *b*-value: 1000 s/mm^2^, and 25 collinear directions with five non-weighted images.

Each DW-MRI image was run through a series of quality assurance tests for artifacts common to DW-MRI, including ghost and/or ring artifact, slice wise intensity-related artifact, venetian blind effect, and gradient-wise motion artifact (Liu et al., 2010). After passing this inspection, the diffusion image was corrected for distortion induced by the gradient coils and simple head motion using the eddy correction routine within FSL (FMRIB Software Library^2^). Following this, Camino (Cook et al., 2006) was used to fit the diffusion tensor, using a positive constraint, and estimate FA at each voxel.

#### PET-derived raphe atlas

In a separate study (unrelated to SSRI treatment), 52 healthy controls were previously scanned using PET and the tracer [^11^C]WAY-100635, which targets the 5-HT_1A_ receptor (Parsey et al., 2010). Voxel binding maps were calculated, warped to a standard image space, and averaged (Parsey et al., 2010). (Each subject’s MRI was first non-linearly warped using the symmetric normalization routine in the Advanced Normalization Tools, ANTs (Avants et al., 2008), to a high-resolution template (Holmes et al., 1998). The resulting non-linear transform was then used to bring each subject’s voxel map into the template space). Since 5-HT_1A_ binding is higher in the raphe nuclei than surrounding regions, a thresholding technique was used to extract the nuclei from the average voxel image. This extracted raphe could be applied to any subject’s MRI by warping the subject’s MRI to the template space, and applying the inverse warp to the raphe template.

#### Seed region

Each anatomical MRI was non-linearly warped to the subject’s corresponding field map-corrected DW-MRI image using ANTs. With the calculated transformation, Freesurfer ROIs and the template-based raphe nuclei (both in anatomical MRI space) could be non-linearly warped to DW-MRI space. The freesurfer-delineated midbrain ROI was then merged with the raphe nuclei extracted from the PET template to ensure that the combined region fully encompassed the raphe, and this combined region was used as a seed for the tractography. The midbrain was used in conjunction with the raphe because the resolution of 25-direction DW-MRI prevented accurate quantification of tracts from the raphe alone.

#### Tractography and weighted FA

Probabilistic tractography was performed using FMRIB’s Diffusion Toolbox (FDT)^3^. This algorithm computes probabilistic streamlines through each voxel by repetitive sampling from the principal diffusion directions. The result of this procedure is the probability of connections from the seed (midbrain plus raphe, defined above) to either the amygdala or the hippocampus. The algorithm was run with a tract curvature threshold of 0.2 mm, maximum number of steps per sample equal to 2000, length of each step equal to 0.5 mm, and 5000 samples. (Note that the resulting tract number is dependent on these initialization parameters, and therefore may only be useful for relative comparisons.) In order to calculate the (weighted) average FA within the defined tracts, the voxel-based FA measures were multiplied by the probability of connection at each voxel and divided by the sum of the probabilities.

## Results

### Tractography

**Figure 1:**
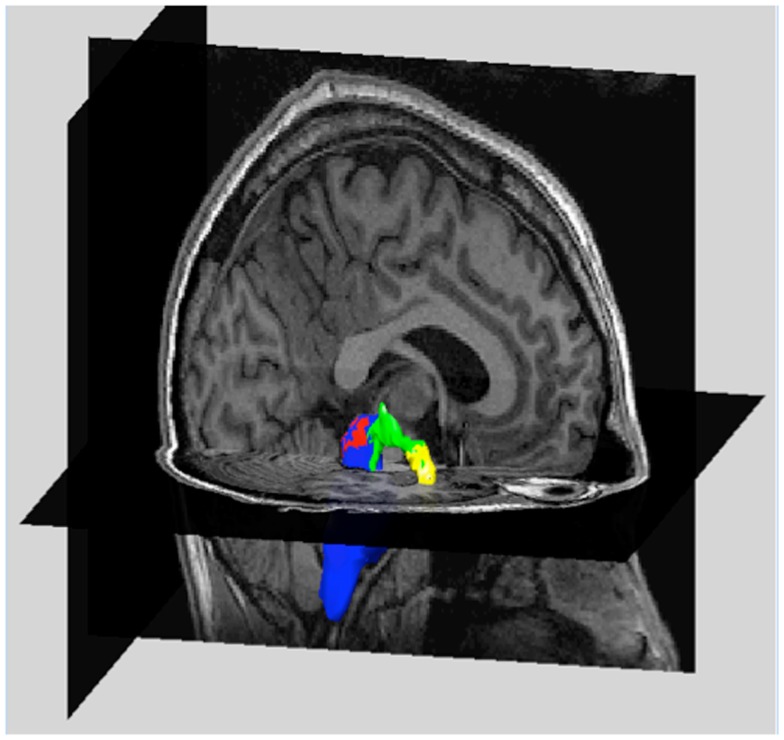
**Example of tractography results in one subject**. The magnetic resonance image (MRI) of one subject is shown. (Cutaway views of a sagittal and axial slice). Overlaid on the MRI are volume renderings of the midbrain (blue) containing the raphe nuclei (red), the right amygdala (yellow), and the tracts between these regions (green). At each voxel, the number of tracts between midbrain and right amygdala were determined and used as weighting factors in the calculation of average fractional anisotropy. Only voxels containing more than 40 tracts are shown.

Tractography results for one subject (right amygdala target) are shown in Figure 1. As indicated in this Figure, tracts can be visualized to ensure that they meaningfully connect seed and target regions. In this figure, only voxel connections containing greater than 40 tracts per voxel are shown.

### Connectivity to amygdala

**Figure 2:**
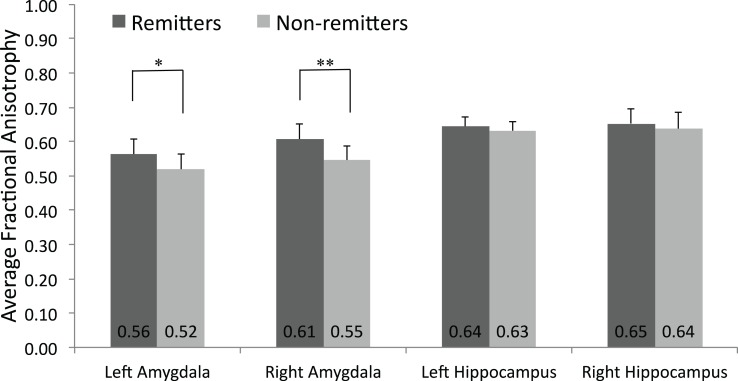
**Mean fractional anisotropy values in the amygdala and hippocampus**. DW-MRI images of were acquired of 18 depressed subjects prior to SSRI treatment. The mean and standard deviation of those who remitted to treatment (*n* = 8) and those who did not (*n* = 10) is plotted.*p = 0.06, **p = 0.007.

Average FA in fiber tracts (weighted by the connection probability) to the right amygdala target (FA_right_amy_) was significantly lower in non-remitters (0.55 ± 0.04) than remitters (0.61 ± 0.04, *p* < 0.01; see Figure 2). At a trend level, the average FA in tracts to the left amygdala target (FA_left_amy_) was also lower in non-remitters (0.52 ± 0.05) versus remitters (0.56 ± 0.04, *p* = 0.06).

The total number of tracts generated between seed and target varied substantially by subject. Therefore, to stabilize variances and correct for any skew in the data, the log transform of the total number of tracts to each target was computed. Using the log-transformed values, on average, non-remitters had fewer tracts to the right (3.18 ± 0.81) and left (3.12 ± 0.58) amygdala compared to remitters (3.91 ± 0.74, *p* = 0.06 and 3.96 ± 0.63, *p* = 0.01, respectively).

### Connectivity to hippocampus

In contrast to the amygdala findings, no significant differences between the response groups were found in average FA values to the right (FA_right_hip_) or left hippocampus target (FA_left_hip_, see Figure 2) or in number of tracts to these targets.

### Continuous measure of treatment outcome

**Figure 3:**
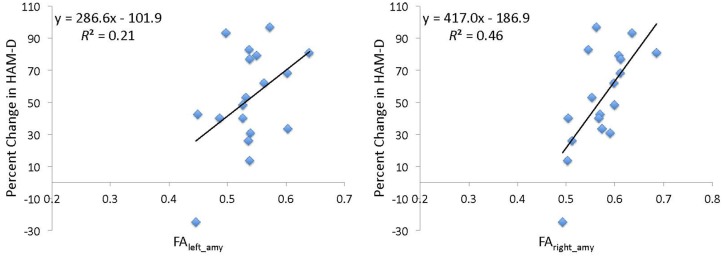
**Percent change in Hamilton depression scale score (HAM-D) as a function of pre-treatment average fractional anisotropy**. Average fractional anisotropy (FA) was calculated using a weighted mean of FA within the tracts from the midbrain/raphe to the left (FA_left_amy_) or right (FA_right_amy_) amygdala. The linear regression fits (black lines) and parameters are indicated.

In addition to being classified as remitter or non-remitter, a subject’s percent change in depression score, defined as 100 × (HAM-D_baseline_−HAM-D_post-treatment_)/HAM-D_baseline_, could also be calculated. Percent change in HAM-D was considered rather than absolute change to account for the effect of the subject’s baseline value on their potential change in HAM-D score. Using regression analysis, FA_right_amy_ predicted percent change in depression scores (*b* = 417.0, *t* = 3.68, *df* = 17, *R^2^* = 0.46, *p* = 0.002, Figure 3). Using the same analysis, FA_left_amy_ had less predictive power (*b* = 286.6, *t* = 2.05, *df* = 17, *R^2^* = 0.21, *p* = 0.06). Adding age and total brain volume to the model did not improve the predictive power of either FA_right_amy_ (*b* = 193.6, *t* = 1.15, *df* = 17, *R^2^* = 0.08, *p* = 0.27) or FA_left_amy_ (*b* = 108.1, *t* = 0.38, *df* = 17, *R^2^* = 0.01, *p* = 0.71).

Since there is one subject whose depression worsened after treatment (Figure 3), the regression analysis was repeated with this subject removed. Without this subject, the FA_right_amy_ finding remained (*b* = 314.7, *t* = 2.89, *df* = 16, *R^2^* = 0.36, *p* = 0.01); however the FA_left_amy_ finding was diminished (*b* = 134.4, *t* = 0.96, *df* = 16, *R^2^* = 0.06, *p* = 0.35).

### Correlation to 5-HT binding measures as assessed by PET

Since this study was motivated by PET results, a preliminary analysis of the relationship between amygdala 5-HT_1A_ binding and FA was performed. For this analysis, 5-HT_1A_ binding was quantified using the PET tracer[^11^C]WAY-100635 (Mathis et al., 1994) and the outcome measure BP_F_, equivalent to *B*_avail_/*K*_D_, where *B*_avail_ is the density of available receptors and *K*_D_ is the equilibrium dissociation constant (Parsey et al., 2000; Innis et al., 2007). Since [^11^C]WAY-100635 binding may be affected by sex, medication status, and genotype (functional C-1019G promoter polymorphism of the 5-HT_1A_ gene; Lemonde et al., 2003), these variables were used as covariates in the regression of raphe BP_F_ versus average FA (Miller et al., 2009). Of the 18 MDD subjects, the frequency of each genotype was: CG: 10 (55.6%); CC: 4 (22.2%); GG: 3 (16.7%); and unknown in one subject (5.6%). In addition, observations were weighted using estimates of BP_F_ measurement precision based on errors estimated by a bootstrapping algorithm (Ogden and Tarpey, 2006; Miller et al., 2012a). No significant correlation was found between left or right amygdala 5-HT_1A_ binding and average FA_left_amy_ or FA_right_amy_ (*b* = −1.14, *t* = −0.02, *df* = 17, *R^2^* = 0.25, *p* = 0.99 and *b* = −23.81, *t* = −0.36, *df* = 17, *R^2^* = 0.29, *p* = 0.73, for left and right respectively). Similarly, no significant correlation was found between left or right amygdala 5-HT_1A_ binding and the log of the total number of tracts to the left or right amygdala (*b* = 5.12, *t* = 1.18, *df* = 17, *R^2^* = 0.26, *p* = 0.26 and *b* = 2.77, *t* = 0.63, *df* = 17, *R^2^* = 0.31, *p* = 0.54, for left and right respectively).

Due to pre-treatment 5-HTT binding differences in the amygdala of remitters versus non-remitters observed in a previous cohort (Miller et al., 2008), the relationship of amygdala 5-HTT binding to average FA was also evaluated. Serotonin transporter binding was assessed using the PET tracer [^11^C]DASB (Houle et al., 2000) and the outcome measure *V*_T_/*f*_p_, where *V*_T_ is the volume of distribution (or ratio of the concentration of the ligand in the region to that in the plasma at equilibrium) and *f*_p_ is the free fraction (Innis et al., 2007; Mukhin et al., 2008; Ichise, 2009; Esterlis et al., 2010; Chin et al., 2011; Fujita et al., 2012). For this analysis, only 17 subjects could be used, because one subject did not have [^11^C]DASB scan data available. As above, observations were weighted using estimates of V_T_/f_p_ measurement precision, based on errors estimated by a bootstrapping algorithm (although no covariates were used in this case; Ogden and Tarpey, 2006; Miller et al., 2012a). No correlation was found between *V*_T_/*f*_p_ in the left or right amygdala and FA_left_amy_ or FA_right_amy_ (*b* = −289.47, *t* = −1.02, *df* = 16, *R^2^* = 0.06, *p* = 0.33 and *b* = 329.79, *t* = 1.16, *df* = 16, *R^2^* = 0.08, *p* = 0.27, for left and right respectively). Similarly, no correlation was found between *V*_T_/*f*_p_ and the log of the total number of tracts (*b* = −12.24, *t* = −0.58, *df* = 16, *R^2^* = 0.02, *p* = 0.57 and *b* = 4.86, *t* = 0.29, *df* = 16, *R^2^* = 0.01, *p* = 0.78, for left and right respectively).

## Discussion

This pilot study evaluated DW-MRI-derived tracts between the midbrain/raphe and the amygdala or hippocampus. In previous PET studies performed by our group, 5-HT_1A_ raphe and 5-HTT amygdala binding has been associated with antidepressant treatment outcome in MDD. This study was an initial attempt to further probe these observed differences, and to determine whether DW-MRI-derived measures can be used as a potential predictor of SSRI antidepressant response in MDD.

Previous PET findings indicated lower pre-treatment 5-HTT binding in the amygdala (as well as lower 5-HT_1A_ binding in the raphe, the region from which most serotonergic neurons originate; Miller et al., 2012a) in non-remitters compared with remitters (Miller et al., 2008). Consistent with these findings, in this study, significantly lower FA_right_amy_ (in tracts originating from the midbrain/raphe) was observed in non-remitters. In addition to lower average FA, the number of tracts to the right amygdala was lower (at a trend level) in non-remitters versus remitters. Although both FA_left_amy_ and number of tracts to the left amygdala were also lower in non-remitters, the effect was stronger on the right side. Similar findings were reported by a recent study using comparable methodology (examination of FA within white matter tracts from the subgenual anterior cingulate to the amygdala or supragenual cingulate). In that study, a significant difference in FA was observed between depressed adolescents and controls in the tracts from the right subgenual anterior cingulate to the right amygdala only (Cullen et al., 2010). Further investigation of possible laterality in FA differences in depression and as a function of treatment outcome is therefore warranted. (It should be noted that, in the PET study, left and right amygdala were combined (Miller et al., 2008)). Interestingly, the Cullen et al. study found FA differences using a different tractography seed (subgenual anterior cingulate). This may suggest that the FA differences observed in the current study are a consequence (and not a cause) of primary pathology in the amygdala. Since causation cannot be determined from this pilot study, this remains an open question.

The hippocampus is another region that has been implicated in depression pathophysiology; however, we have not previously found a significant correlation between serotonin receptor or transporter binding in the hippocampus and antidepressant treatment outcome. Correspondingly, no significant differences in FA or number of tracts to the left or right hippocampus between non-remitters and remitters were observed. This lends further credence to the potential interrelation between serotonin findings and white matter connectivity, as assessed by DW-MRI. However, due to the small sample size and limitations listed below, a relationship between FA in tracts to the hippocampus and remission status cannot be ruled out without further studies in a larger cohort.

Since most serotonin fibers originate from the raphe, one potential explanation based on the DW-MRI findings in combination with those from PET, is that subjects who remit to antidepressant treatment have greater health and/or number of serotonergic neurons originating in the raphe and terminating in the amygdala. In this case, one might expect to see a correlation between FA (or the number of tracts) and 5-HT or 5-HT_1A_ binding; however, these correlations were not statistically significant in this work. This may have been due to the small sample size and/or the need for multiple PET covariates (or other limitations discussed in 4.1). Lending credence to this hypothesis, the statistically significant difference in PET binding measures between remitters and non-remitters is not replicated in sample of subjects who received DW-MRI imaging (data not shown). (Although it should be noted, that in addition to the reduced sample size, the left and right amygdala were also considered separately in this work, adding to variability in the binding measurement). In addition, it is possible that a correlation was not observed due to the heterogeneity of depression itself. Since the etiology of depression is not known completely, depression pathophysiology, as well as potential downstream antidepressant effects are not fully elucidated. The monoaminergic system, glutamate, neurotrophic factors, and circadian rhythms have all been implicated in MDD (Hasler, 2010). Therefore the relationship between PET-based serotoninergic binding measures, FA, and clinical outcomes may be much more complex than can be explained through a simple correlation analysis. Future studies will be needed to address these issues.

To further investigate the sensitivity of DW-MRI-derived measures to an individual’s SSRI treatment response, linear regression analysis was performed. This analysis (Figure 3) indicates that pre-treatment FA_right_amy_ predicts some of the variance in the degree of clinical improvement following SSRI treatment. One potential interpretation is that the reduced regional white matter connectivity (observed in non-remitters) can impair effectiveness of antidepressant treatments, resulting in reduced depression improvement. Further DW-MRI studies are needed to validate this hypothesis. Examining the tracts to the left amygdala reveals that FA_left_amy_ provides less predictive power of final depression scores, especially when the single subject whose depression worsens is removed. Similar to the FA results, this also suggests a stronger effect on the right side.

This was a pilot study, and potentially the first to use DW-MRI to predict antidepressant treatment response. As such, there were a limited number of subjects examined (*n* = 18), and these subjects were further divided into remitters and non-remitters based on treatment response. Because of this, to avoid the loss of statistical power related to multiple comparisons, the scope of this work was restricted to the evaluation of connectivity between the midbrain/raphe (from which most serotonergic fibers originate) and two brain regions – the amygdala and hippocampus. The main finding of this work is therefore related to the examination of the average FA between these two groups. Although further analysis (i.e., prediction of final Hamilton Depression Score or relation to serotonin binding) was performed to aid in the interpretation of the main finding, further detailed analysis was challenging due to the following limitations.

### Limitations

(1) Interpretation of DW-MRI results. Recent publications have highlighted several issues related to DW-MRI interpretation (Jones, 2010; Jones et al., 2012). There is remaining uncertainty over the optimal method to reconstruct tracts from diffusion-weighted images, and choice of software and underlying model assumptions may greatly affect results (Fillard et al., 2011). Although the probabilistic method used in this work has been partially validated (Behrens et al., 2003) and is designed to handle multiple fiber orientations (Behrens et al., 2007), novel applications of this technique (such as fiber tracking between the raphe and amygdala) require further validation. (2) Anatomical validation. To fully interpret DW-MRI results, it is essential to validate DW-MRI-derived results anatomically, which was not performed as part of this pilot study (Jones, 2010). Since a “tract” is not derived from a single neuron, it may represent a fiber bundle, or multiple fiber bundles (Catani et al., 2002). It is therefore necessary to validate that tractography results represent the serotonergic pathway and not, for example, the more prevalent dopaminergic pathway or a combination of fibers. (Although, when searching for a clinically relevant biomarker of antidepressant treatment effectiveness, it is only necessary to distinguish between groups). (3) The resolution of 25-direction DW-MRI. The resolution of DW-MRI is determined, in large part, by the number of gradient directions measured. The limited resolution of 25-direction DW-MRI may have led to variability in the tractography results and prevented using the small raphe region as a seed. Because of this, the midbrain (including the raphe) was used. However, this is unlikely to have a great effect since, fibers from the midbrain that terminate at the amygdala or hippocampus will most likely originate in the raphe.

Despite the concerns listed above, the significant differences observed in FA_right_amy_ between treatment groups indicate that it is a robust finding and, as such, that DW-MRI-derived measures may provide a pre-treatment indicator of antidepressant response.

## Conclusion

Selection of antidepressant treatment is a challenge for both clinicians and patients. This pilot study was performed to determine the potential of DW-MRI-derived measures to provide a pre-treatment indicator of SSRI response. A significant difference was observed in FA_right_amy_ (average FA within white matter tracts between the raphe and the right amygdala) between SSRI remitters and non-remitters. Moreover, there was a significant correlation between FA_right_amy_ and percent improvement in depression, as assessed by the Hamilton Depression Rating Scale (HAM-D). These results suggest that the health and/or number of serotonergic fibers terminating at the right amygdala may be compromised in SSRI non-remitters. Moreover, since these differences were less robust in tracts terminating at the left amygdala and not observed in tracts terminating at the hippocampus, the observed effect may be localized regionally. However, due to limitations in DW-MRI resolution and sample size, future work in a larger cohort is required to confirm these findings. These initial findings do suggest, however, that DW-MRI-based measures should be further investigated as a potential pre-treatment signature of antidepressant response.

## Conflict of Interest Statement

The authors declare that the research was conducted in the absence of any commercial or financial relationships that could be construed as a potential conflict of interest.

## Footnotes

^1^http://surfer.nmr.mgh.harvard.edu/

^2^http://www.fmrib.ox.ac.uk/fsl/

^3^http://www.fmrib.ox.ac.uk/fsl/fdt/fdt_probtrackx.html

## References

[B1] Avants B. B. Epstein C. L. Grossman M. Gee J. C. (2008). Symmetric diffeomorphic image registration with cross-correlation: evaluating automated labeling of elderly and neurodegenerative brain. Med. Image Anal. 12, 26–41 10.1016/j.media.2007.06.004 17659998 PMC2276735

[B2] Beaulieu C. (2002). The basis of anisotropic water diffusion in the nervous system – a technical review. NMR Biomed. 15, 435–455 10.1002/nbm.782 12489094

[B3] Behrens T. E. Berg H. J. Jbabdi S. Rushworth M. F. Woolrich M. W. (2007). Probabilistic diffusion tractography with multiple fibre orientations: what can we gain? Neuroimage 34, 144–155 10.1016/j.neuroimage.2006.09.018 17070705 PMC7116582

[B4] Behrens T. E. Woolrich M. W. Jenkinson M. Johansen-Berg H. Nunes R. G. Clare S. (2003). Characterization and propagation of uncertainty in diffusion-weighted MR imaging. Magn. Reson. Med. 50, 1077–1088 10.1002/mrm.10609 14587019

[B5] Catani M. Howard R. J. Pajevic S. Jones D. K. (2002). Virtual in vivo interactive dissection of white matter fasciculi in the human brain. Neuroimage 17, 77–94 10.1006/nimg.2002.1136 12482069

[B6] Chin C. L. Carr R. A. Llano D. A. Barret O. Xu H. Batis J. (2011). Pharmacokinetic modeling and [(1)(2)(3)]5-IA-85380 single photon emission computed tomography imaging in baboons: optimization of dosing regimen for ABT-089. J. Pharmacol. Exp. Ther. 336, 716–723 10.1124/jpet.110.173880 21172907

[B7] Cook P. A. Bai Y. Nedjati-Gilani S. Seunarine K. K. Hall M. G. Parker G. J. (2006). Camino: open-source diffusion-mri reconstruction and processing. 14th Scientific Meeting of the International Society for Magnetic Resonance in Medicine, Seattle, 2759

[B8] Cullen K. R. Klimes-Dougan B. Muetzel R. Mueller B. A. Camchong J. Houri A. (2010). Altered white matter microstructure in adolescents with major depression: a preliminary study. J. Am. Acad. Child Adolesc. Psychiatry 49, 173–183e1. 10.1016/j.jaac.2009.11.005 20215939 PMC2909686

[B9] Esterlis I. Cosgrove K. P. Batis J. C. Bois F. Stiklus S. M. Perkins E. (2010). Quantification of smoking-induced occupancy of beta2-nicotinic acetylcholine receptors: estimation of nondisplaceable binding. J. Nucl. Med. 51, 1226–1233 10.2967/jnumed.109.072447 20660383 PMC3707518

[B10] Fillard P. Descoteaux M. Goh A. Gouttard S. Jeurissen B. Malcolm J. (2011). Quantitative evaluation of 10 tractography algorithms on a realistic diffusion MR phantom. Neuroimage 56, 220–234 10.1016/j.neuroimage.2011.01.032 21256221

[B11] Fujita M. Hines C. S. Zoghbi S. S. Mallinger A. G. Dickstein L. P. Liow J. S. (2012). Downregulation of brain phosphodiesterase type IV measured with (11)C-(R)-rolipram positron emission tomography in major depressive disorder. Biol. Psychiatry 72, 548–554 10.1016/j.biopsych.2012.04.030 22677471 PMC3438357

[B12] Hasler G. (2010). Pathophysiology of depression: do we have any solid evidence of interest to clinicians? World Psychiatry 9, 155–161 20975857 10.1002/j.2051-5545.2010.tb00298.x PMC2950973

[B13] Holmes A. (2008). Genetic variation in cortico-amygdala serotonin function and risk for stress-related disease. Neurosci. Biobehav. Rev. 32, 1293–1314 10.1016/j.neubiorev.2008.03.006 18439676 PMC2561331

[B14] Holmes C. J. Hoge R. Collins L. Woods R. Toga A. W. Evans A. C. (1998). Enhancement of MR images using registration for signal averaging. J. Comput. Assist. Tomogr. 22, 324–333 10.1097/00004728-199803000-00032 9530404

[B15] Houle S. Ginovart N. Hussey D. Meyer J. H. Wilson A. A. (2000). Imaging the serotonin transporter with positron emission tomography: initial human studies with [11C]DAPP and [11C]DASB. Eur. J. Nucl. Med. 27, 1719–1722 10.1007/s002590000365 11105830

[B16] Ichise M. (2009). “Neuroreceptor Imaging and Kinetic Modeling,” in Functional Cerebral SPECT and PET Imaging, 4th Edn, eds Van Heertum R. L. Tikofsky R. S. Ichise M. (Philadelphia, PA: Lippincott Williams & Wilkins), 44

[B17] Innis R. B. Cunningham V. J. Delforge J. Fujita M. Gjedde A. Gunn R. N. (2007). Consensus nomenclature for in vivo imaging of reversibly binding radioligands. J. Cereb. Blood Flow Metab. 27, 1533–1539 10.1038/sj.jcbfm.9600493 17519979

[B18] Jabeen Haleem D. (2011). Raphe-hippocampal serotonin neurotransmission in the sex related differences of adaptation to stress: focus on serotonin-1A receptor. Curr. Neuropharmacol. 9, 512–521 10.2174/157015911796558019 22379463 PMC3151603

[B19] Jasinska A. J. Lowry C. A. Burmeister M. (2012). Serotonin transporter gene, stress and raphe-raphe interactions: a molecular mechanism of depression. Trends Neurosci. 35, 395–402 10.1016/j.tins.2012.01.001 22301434

[B20] Jones D. K. (2010). Challenges and limitations of quantifying brain connectivity in vivo with diffusion MRI. Imaging Med. 2, 341–355 10.2217/iim.10.21

[B21] Jones D. K. Knosche T. R. Turner R. (2012). White matter integrity, fiber count, and other fallacies: the do’s and don’ts of diffusion MRI. NeuroImage. 10.1016/j.neuroimage.2012.06.081 22846632

[B22] Kirino E. (2012). Escitalopram for the management of major depressive disorder: a review of its efficacy, safety, and patient acceptability. Patient Prefer. Adherence 6, 853–861 23271894 10.2147/PPA.S22495 PMC3526882

[B23] LeDoux J. (2003). The emotional brain, fear, and the amygdala. Cell. Mol. Neurobiol. 23, 727–738 10.1023/A:1025048802629 14514027 PMC11530156

[B24] Lemonde S. Turecki G. Bakish D. Du L. Hrdina P. D. Bown C. D. (2003). Impaired repression at a 5-hydroxytryptamine 1A receptor gene polymorphism associated with major depression and suicide. J. Neurosci. 23, 8788–8799 14507979 10.1523/JNEUROSCI.23-25-08788.2003 PMC6740417

[B25] Leuchter A. F. Cook I. A. Hamilton S. P. Narr K. L. Toga A. Hunter A. M. (2010). Biomarkers to predict antidepressant response. Curr. Psychiatry Rep. 12, 553–562 10.1007/s11920-010-0160-4 20963521 PMC2965366

[B26] Leuchter A. F. Lesser I. M. Trivedi M. H. Rush A. J. Morris D. W. Warden D. (2008). An open pilot study of the combination of escitalopram and bupropion-SR for outpatients with major depressive disorder. J. Psychiatr. Pract. 14, 271–280 10.1097/01.pra.0000336754.19566.65 18832958 PMC2778329

[B27] Liu Z. Wang Y. Gerig G. Gouttard S. Tao R. Fletcher T. (2010). “Quality control of diffusion tensor images,” in Proceedings of the SPIE, San Diego, CA, 1–9 10.1117/12.844748 PMC3864968 24353379

[B28] Lopez J. F. Chalmers D. T. Little K. Y. Watson S. J. (1998). A. E. Bennett research award. Regulation of serotonin1A, glucocorticoid, and mineralocorticoid receptor in rat and human hippocampus: implications for the neurobiology of depression. Biol. Psychiatry 43, 547–573 10.1016/S0006-3223(98)90581-9 9564441

[B29] Lowry C. A. Hale M. W. Evans A. K. Heerkens J. Staub D. R. Gasser P. J. (2008). Serotonergic systems, anxiety, and affective disorder: focus on the dorsomedial part of the dorsal raphe nucleus. Ann. N. Y. Acad. Sci. 1148, 86–94 10.1196/annals.1410.004 19120094

[B30] Mathis C. A. Simpson N. R. Mahmood K. Kinahan P. E. Mintun M. A. (1994). [11C]WAY 100635: a radioligand for imaging 5-HT1A receptors with positron emission tomography. Life Sci. 55, PL403–PL407 10.1016/0024-3205(94)00324-6 7968222

[B31] McEwen B. S. Magarinos A. M. (2001). Stress and hippocampal plasticity: implications for the pathophysiology of affective disorders. Hum. Psychopharmacol. 16, S7–S19 10.1002/hup.266 12404531

[B32] Meltzer H. Y. (1990). Role of serotonin in depression. Ann. N. Y. Acad. Sci. 600, 486–499; discussion 499–500. 10.1111/j.1749-6632.1990.tb16904.x 2252328

[B33] Miller J. M. Brennan K. G. Ogden T. R. Oquendo M. A. Sullivan G. M. Mann J. J. (2009). Elevated serotonin 1A binding in remitted major depressive disorder: evidence for a trait biological abnormality. Neuropsychopharmacology 34, 2275–2284 10.1038/npp.2009.54 19458612 PMC2760406

[B34] Miller J. M. Hesselgrave N. Ogden R. T. Mann J. J. Parsey R. V. (2012a). Brain serotonin 1A receptor predicts treatment outcome in major depressive disorder. Biol. Psychiatry [submitted]. 10.1016/j.biopsych.2012.02.034

[B35] Miller J. M. Hesselgrave N. Ogden R. T. Oquendo M. A. Mann J. J. Parsey R. V. (2012b). Elevated Serotonin 1A Receptor Binding in Raphe Nuclei is Associated with Remission to the Antidepressant Escitalopram. Philadelphia, PA: Society of Biological Psychiatry

[B36] Miller J. M. Oquendo M. A. Ogden R. T. Mann J. J. Parsey R. V. (2008). Serotonin transporter binding as a possible predictor of one-year remission in major depressive disorder. J. Psychiatr. Res. 42, 1137–1144 10.1016/j.jpsychires.2008.01.012 18331740 PMC2670200

[B37] Mukhin A. G. Kimes A. S. Chefer S. I. Matochik J. A. Contoreggi C. S. Horti A. G. (2008). Greater nicotinic acetylcholine receptor density in smokers than in nonsmokers: a PET study with 2-18F-FA-85380. J. Nucl. Med. 49, 1628–1635 10.2967/jnumed.108.050716 18794265 PMC2766917

[B38] Murphy M. L. Frodl T. (2011). Meta-analysis of diffusion tensor imaging studies shows altered fractional anisotropy occurring in distinct brain areas in association with depression. Biol. Mood Anxiety Disord. 1:3 10.1186/2045-5380-1-3 22738088 PMC3377129

[B39] Nobuhara K. Okugawa G. Sugimoto T. Minami T. Tamagaki C. Takase K. (2006). Frontal white matter anisotropy and symptom severity of late-life depression: a magnetic resonance diffusion tensor imaging study. J. Neurol. Neurosurg. Psychiatr. 77, 120–122 10.1136/jnnp.2004.055129 16361611 PMC2117392

[B40] Ogden R. T. Ojha A. Erlandsson K. Oquendo M. A. Mann J. J. Parsey R. V. (2007). In vivo quantification of serotonin transporters using [(11)C]DASB and positron emission tomography in humans: modeling considerations. J. Cereb. Blood Flow Metab. 27, 205–217 10.1038/sj.jcbfm.9600329 16736050 PMC3784003

[B41] Ogden R. T. Tarpey T. (2006). Estimation in regression models with externally estimated parameters. Biostatistics 7, 115–129 10.1093/biostatistics/kxi044 16020616

[B42] Owens M. J. Rosenbaum J. F. (2002). Escitalopram: a second-generation SSRI. CNS Spectr. 7, 34–39 15131491 10.1017/s1092852900028583

[B43] Parsey R. V. Ogden R. T. Miller J. M. Tin A. Hesselgrave N. Goldstein E. (2010). Higher serotonin 1A binding in a second major depression cohort: modeling and reference region considerations. Biol. Psychiatry 68, 170–178 10.1016/j.biopsych.2010.03.023 20497898 PMC2900398

[B44] Parsey R. V. Slifstein M. Hwang D. R. Abi-Dargham A. Simpson N. Mawlawi O. (2000). Validation and reproducibility of measurement of 5-HT1A receptor parameters with [carbonyl-11C]WAY-100635 in humans: comparison of arterial and reference tisssue input functions. J. Cereb. Blood Flow Metab. 20, 1111–1133 10908045 10.1097/00004647-200007000-00011

[B45] Ressler K. J. Nemeroff C. B. (2000). Role of serotonergic and noradrenergic systems in the pathophysiology of depression and anxiety disorders. Depress. Anxiety 12(Suppl. 1), 2–19 10.1002/1520-6394(2000)12:1+<2::AID-DA2>3.0.CO;2-4 11098410

[B46] Sexton C. E. Mackay C. E. Ebmeier K. P. (2009). A systematic review of diffusion tensor imaging studies in affective disorders. Biol. Psychiatry 66, 814–823 10.1016/j.biopsych.2009.05.024 19615671

[B47] Taylor C. B. Youngblood M. E. Catellier D. Veith R. C. Carney R. M. Burg M. M. (2005). Effects of antidepressant medication on morbidity and mortality in depressed patients after myocardial infarction. Arch. Gen. Psychiatry 62, 792–798 10.1001/archpsyc.62.7.792 15997021

[B48] Tenke C. E. Kayser J. Gates N. A. Alschuler D. M. Kroppmann C. J. Fekri S. (2010). “Auditory evoked potential (AEP) and EEG measures in depressed patients predict response to antidepressants. Symposium Presentation at the 65th Society of Biol Psychiatry, New Orleans, LA

